# Multi-symptom asthma is closely related to nasal blockage, rhinorrhea and symptoms of chronic rhinosinusitis-evidence from the West Sweden Asthma Study

**DOI:** 10.1186/1465-9921-11-163

**Published:** 2010-11-26

**Authors:** Jan Lötvall, Linda Ekerljung, Bo Lundbäck

**Affiliations:** 1 Krefting Research Centre, Sahlgrenska Academy, University of Gothenburg, Sweden

## Abstract

**Background:**

We have previously shown that approximately 25% of those with asthma in West Sweden have multiple asthma symptoms, which may describe a group of patients with more severe disease. Furthermore, asthma is associated with several co-morbid diseases, including rhinitis and chronic rhinosinusitis. The aim of this study was to determine whether multi-symptom asthma is related to signs of severe asthma, and to investigate the association between multi-symptom asthma and different symptoms of allergic and chronic rhinosinusitis.

**Methods:**

This study analyzed data on asthma symptoms, rhinitis, and chronic rhinosinusitis from the 2008 West Sweden Asthma Study, which is an epidemiologically based study using the OLIN and GA^2^LEN respiratory and allergy focused questionnaires.

**Results:**

Multi-symptom asthma was present in 2.1% of the general population. Subjects with multi-symptom asthma had more than double the risk of having night-time awakenings caused by asthma compared with those with fewer asthma symptoms (P < 0.001). The prevalence of allergic rhinitis was similar in the fewer- and multi-symptom asthma groups, but nasal blockage and rhinorrhea were significantly increased in those with multi- versus fewer-symptom asthma (odds ratio 2.21; 95% confidence interval 1.64-2.97, versus 1.49; 1.10-2.02, respectively). Having any, or one to four symptoms of chronic rhinosinusitis significantly increased the risk of having multi- versus fewer-symptom asthma (P < 0.01).

**Conclusion:**

An epidemiologically identified group of individuals with multiple asthma symptoms harbour to greater extent those with signs of severe asthma. The degree of rhinitis, described by the presence of symptoms of nasal blockage or rhinorrhea, as well as the presence of any or several signs of chronic rhinosinusitis, significantly increases the risk of having multi-symptom asthma.

## Background

Asthma is a common chronic disease with a prevalence of approximately 5-10% in different populations [1-6]. We have recently shown that the prevalence of asthma in West Sweden is approximately 8.5%, based on a large epidemiological survey [6]. Importantly, our data argue that there has been no further increase in the prevalence of asthma over the last 18 years in this part of Europe, and moreover that the overall degree of airway symptoms have decreased over this period [6]. However, in the current survey we identify a large population of individuals with multiple asthma symptoms, which amounts to approximately 25% of all asthmatics, and 2% of the general population [6].

Asthma is associated with several co-morbid diseases, including rhinitis and chronic rhino-sinusitis. Several studies have shown a relationship between nasal symptoms and asthma, and rhinitis is identified as an important risk factor of developing asthma [7-10]. Furthermore, studies that have recruited asthma patients from different clinical cohorts have shown that severity of nasal symptoms is associated with severity and difficulty to treat the asthma [9,11-16]. Despite these findings, no epidemiological studies have described the relationship between different nasal symptoms and asthma symptoms in a large random population sample. Furthermore, multi-symptom asthma, identifiable by epidemiological means, has not been described previously.

The aim of the current study was to determine whether multi-symptom asthma is related to signs of severe asthma, and to describe the association between multi-symptom asthma and different symptoms of nasal disease in a general population. In particular, we investigate the relationship between multi-symptom asthma and symptoms of chronic rhinosinusitis, defined as nasal symptoms ongoing beyond 12 weeks a year.

## Methods

### Study population and participation

The study population has been described previously [6]. Briefly, in 2008 a folder containing two questionnaires was mailed out to 30,000 randomly selected subjects, aged 16-75, living in the West of Sweden; 15,000 subjects lived in the urban area of Gothenburg and 15,000 in the remaining region of West Sweden. 29 218 could be traced. The total response rate after three reminders was 62%, and the final study sample consisted of 18 067 subjects. A non-response study performed showed no differences in prevalence of symptoms or disease between responders and non-responders [17].

### Questionnaire

The questionnaires used in the study have been described in detail elsewhere [6]. In brief, the folder contained two questionnaires: 1) the Swedish Obstructive Lung Diseases in Northern Sweden (OLIN) questionnaire [18] with additional questions on work and housing conditions; and 2) the Swedish version of the Global Allergy and Asthma European Network (GA^2^LEN) questionnaire [6]. The questionnaires contained questions on asthma, allergic rhinitis, respiratory and nasal symptoms, use of asthma medication, and possible determinants of the disease.

### Definitions

The definitions in this manuscript are based on the following questions:

*Physician-diagnosed asthma: *"Have you been diagnosed as having asthma by a doctor?"; *Asthma medication: *"Do you currently use asthma medicine (continuously or as needed)?"; *Attacks of shortness of breath: *"Do you presently have, or have you had in the last 10 years, asthma symptoms (intermittent breathlessness or attacks of shortness of breath; the symptoms may exist simultaneously with or without cough or wheezing)?" and "Have you had these symptoms within the last year?"; *Any wheeze: *"Have you had whistling or wheezing in the chest at any occasion during the last 12 months?"; *Recurrent wheeze: *"Do you usually have wheezing or whistling in your chest when breathing?"; *Dyspnea: *"Do you get breathless when you walk on level ground with people of your own age?"; *Breathlessness-exertion: *"Do you usually have breathlessness, wheeze or severe cough on exertion?"; *Breathlessness-cold: *"Do you usually have breathlessness, wheeze or severe cough in cold weather?"; *Breathlessness-exertion in cold: *"Do you usually have breathlessness, wheeze or severe cough on exertion in cold weather?"; *Allergic rhinitis*: "Have you now, or have you ever had, allergic rhinitis (hay-fever) or allergic eye catarrh?"; *Nasal blockage: *"Do you have nasal blockage more or less constantly?"; *Rhinorrhea: *"Do you have a runny nose more or less constantly?"; *Family history of asthma: "*Do any of your parents or sibling have, or have had, asthma?"; *Family history of allergy: "*Do any of your parents or sibling have, or have had, allergic rhinitis or allergic eye catarrh?"; *Occupational exposure: *"Have you been heavily exposed to gas, dust or fumes at work?"; *Waking-cough*: "Have you been woken by an attack of coughing at any time in the last 12 months?"; *Waking-up with shortness of breath*: "Have you been woken by an attack of shortness of breath at any time in the last 12 months?"; *Waking-tight chest: *"Have you woken up with tightness in your chest at any time during the last 12 months?"; *Physician diagnosed chronic sinusitis: "*Has a doctor ever told you that you have chronic sinusitis?"; *Nasal blockage, at least 12 weeks; *"Has your nose been blocked for more than 12 weeks during the last 12 months?".

### Definition of multi-symptom asthma

To be considered having multi-symptom asthma, a subject was required to report *physician-diagnosed asthma *and a*sthma medication *and *attacks of shortness of breath *and *recurrent wheeze *and *at least one out of any wheeze, dyspnoea, breathlessness-exertion, breathlessness-cold and breathlessness-exertion in cold*.

For the purpose of this paper all subjects reporting physician-diagnosed asthma and not fulfilling the requirements of multi-symptom asthma are referred to as having fewer-symptom asthma.

### Ethical approval

The regional ethic committee in West Sweden approved the study.

### Analyses

Statistical analyses were performed using SPSS version 16.0. Comparisons of proportions were tested with a chi-square test or Fisher's exact test. A P-value of <0.05 was regarded as statistically significant. Covariates used in multiple logistic regression analyses were: family history of asthma and/or allergy, smoking habits, age, occupational exposure to gas, dust or fumes, and gender. In addition to these covariates, allergic rhinitis, blocked nose, and runny nose were added one by one and all together. Odds ratios (OR) with 95% confidence intervals (CI) are reported. Logistic regression models were performed in three combinations: non-asthma versus fewer-symptom asthma, non-asthma versus multi-symptom asthma and fewer-symptom asthma versus multi-symptom asthma.

## Results

### Relationship between multi-symptom asthma and night-time asthma symptoms

**Figure 1:**
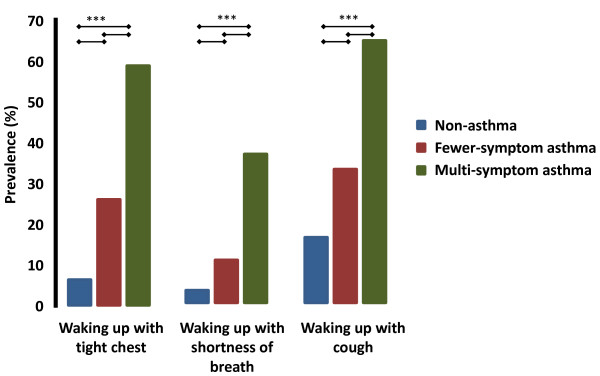
**Prevalence of waking up with tight chest, shortness of breath or cough, during the last 12 months, in the non-asthma, fewer-symptom asthma or multi-symptom asthma groups**. Subjects with multi-symptom asthma had a higher risk of waking up at night regardless of which respiratory symptom is analyzed. Blue: non-asthma, maroon, fewer-symptom asthma and green: multi-symptom asthma. *** P < 0.001.

The subjects that reported multi-symptom asthma (2.1% of the whole population) had a high risk of having night-time awakenings due to chest-tightness, shortness of breath or cough compared with both the populations without asthma and fewer-symptom asthma (P < 0.001, Figure 1).

### Prevalence of allergic rhinitis, nasal blockage and rhinorrhea

Reported allergic rhinitis (AR) was more prevalent among subjects with fewer-symptom asthma (64.4%) and multi-symptom asthma (65.7%) compared with subjects without asthma (22.9%; P < 0.001 in both cases, Table 1). There was no significant difference in the prevalence of reported allergic rhinitis between the populations with fewer-symptom asthma versus the population with multi-symptom asthma (Table 1).

**Table 1:** Prevalence (%) of nasal symptoms by asthma population in the West Sweden Asthma Study (18,087 responders)

		P-values		
		
Exposure	Non- asthma	Non asthma vs. fewer-symptom asthma	Non-asthma vs. multi-symptom asthma	Fewer-symptom asthma vs. multi-symptom asthma
	n=16,380			
**Allergic rhinitis**	22.9	**< 0.001**	**< 0.001**	0.667
**Nasal blockage**	13.1	**< 0.001**	**< 0.001**	**< 0.001**
**Rhinorrhea**	11.6	**< 0.001**	**< 0.001**	**< 0.001**
**Any of the above**	33.3	**< 0.001**	**< 0.001**	**0.002**
**All of the above**	3.5	**< 0.001**	**< 0.001**	**< 0.001**

**Figure 2:**
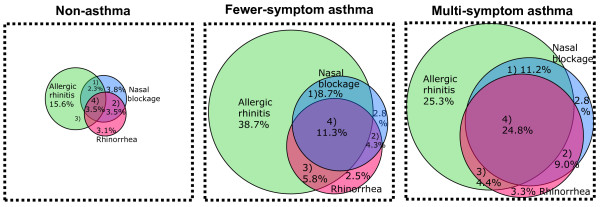
**Venn diagram describing the frequency of reported allergic rhinitis, nasal blockage and/or rhinorrhea in the non-asthma, fewer-symptom asthma and multi-symptom asthma groups**. The diagram illustrates that more subjects in the multi-symptom asthma group have multiple nasal symptoms.

The prevalence of reported nasal blockage and rhinorrhea was higher in the group with multi-symptom asthma compared with the fewer-symptom asthma group (Table 1). Reports of any nasal symptom (AR, nasal blockage or rhinorrhea) occurred in 81.7% of the multi-symptom asthma group, 74.0% of the fewer-symptom asthma group, and 33.3% of the non-asthma population (Table 1). The frequency of nasal symptoms in the non-asthma, fewer-symptom asthma, and multi-symptom asthma groups are shown Figure 2. The prevalence of all three nasal symptoms was higher in subjects with multi-symptom asthma (P < 0.01; Figure 2).

### Multivariate relationships between nasal symptoms and multi-symptom asthma

**Figure 3:**
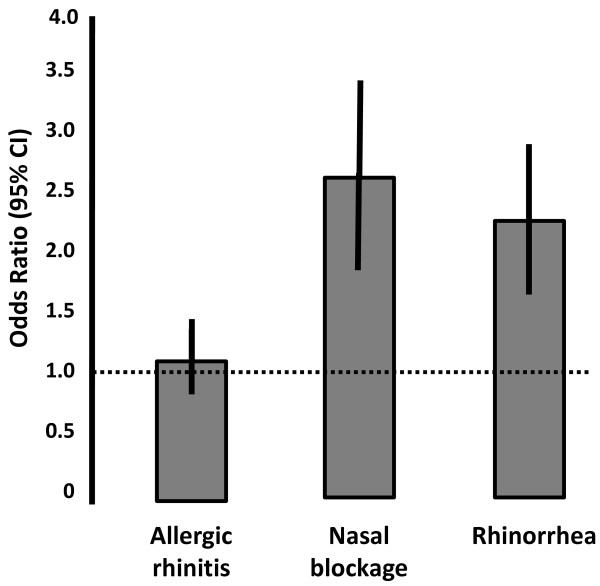
**Odds ratio of having multi-symptom asthma in subjects with reported allergic rhinitis, nasal blockage or rhinorrhea (error bars show 95% confidence intervals)**. Both nasal blockage and rhinorrhea increase the risk of having multi-symptom asthma, however, the presence of allergic rhinitis alone is not a risk factor for multi-symptom asthma.

Nasal blockage and rhinorrhea were strong risk factors for multi-symptom asthma compared with fewer-symptom asthma (OR 2.68 and 2.24, respectively; Figure 3) while reports of allergic rhinitis were not associated with an increased risk of having multi-symptom asthma versus fewer-symptom asthma. In a multiple logistic regression analysis, nasal blockage and rhinorrhea remained statistically significant risk factors, however, with slightly lower ORs (Table 2). Additional risk factors for multi-symptom asthma compared with fewer-symptom asthma in the multiple logistic regression model were: family history of allergy, family history of combined asthma and allergy, old age (> 60 years), occupational exposure to gas, dust or fumes, and female gender (Table 2). In the multiple regression models, comparing multi-symptom asthma and fewer-symptom asthma with non-asthma, AR was the strongest risk factor for fewer-symptom asthma (OR 5.0) and multi-symptom asthma (OR 3.7; Table 2) versus no asthma. Nasal blockage and rhinorrhea were also significant risk factors in these models (Table 2).

**Table 2:** Risk factors, presented as odds ratios (OR) and 95% confidence intervals (CI) for fewer-symptom asthma and multi-symptom asthma from a multiple logistic regression analysis of 18,087 responders in the West Sweden Asthma Study.

		Non-asthma vs. fewer-symptom asthma	Non-asthma vs. multi-symptom asthma	Fewer-symptom asthma vs. multi-symptom asthma
		
Risk factors		OR (95% CI)	OR (95% CI)	OR (95% CI)
**Family history**	+Asthma-Allergy	**2.42 (1.93-3.05)**	**2.44 (1.55-3.84)**	1.15 (0.69-1.91)
	+Allergy-Asthma	0.97 (0.81-1.15)	**1.42 (1.03-1.97)**	**1.53 (1.05-2.22)**
	Both	**2.39 (2.02-2.82)**	**3.68 (2.72-4.99)**	**1.63 (1.16-2.29)**
				
**Smoking**	Ex-smokers	**1.41 (1.22-1.63)**	1.15 (0.87-1.52)	0.76 (0.56-1.05)
	Smokers	1.08 (0.92-1.27)	1.26 (0.96-1.66)	1.28 (0.93-1.77)
				
**Age (years)**	31-45	0.91 (0.78-1.07)	0.97 (0.71-1.33)	1.07 (0.75-1.52)
	46-60	**0.70 (0.59-0.83)**	1.16 (0.84-1.59)	1.70 (1.18-2.45)
	61-75	**0.80 (0.66-0.97)**	**1.70 (1.21-2.38)**	**2.08 (1.40-3.09)**
				
**Occupational exposure**		**1.22 (1.06-1.41)**	**1.63 (1.28-2.07)**	**1.36 (1.03-1.80)**
				
**Gender**	Women	1.12 (0.99-1.27)	**1.59 (1.26-2.02)**	**1.31 (1.00-1.72)**
				
**Allergic rhinitis**		**4.98 (4.37-5.68)**	**3.72 (2.90-4.76)**	0.97 (0.73-1.29)
				
**Nasal blockage**		**1.29 (1.09-1.52)**	**2.82 (2.15-3.71)**	**2.21 (1.64-2.97)**
				
**Rhinorrhea**		**1.31 (1.10-1.55)**	**1.75 (1.32-2.31)**	**1.49 (1.10-2.02)**

All co-variates incorporated in the analysis are presented.

### Symptoms of chronic rhinosinusitis

**Figure 4:**
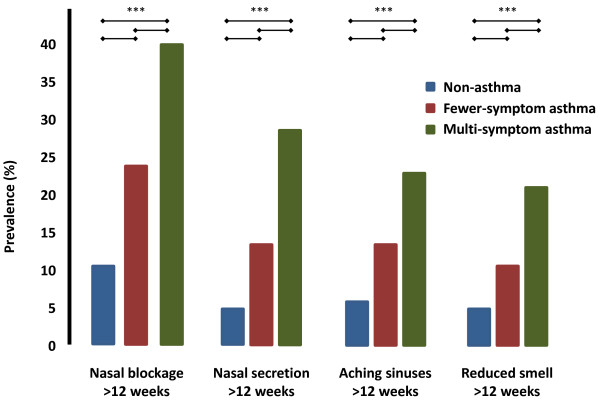
**Frequency of non-asthma, fewer-symptom asthma or multi-symptom asthma in subjects who reported symptoms of chronic rhinosinusitis, including nasal blockage, nasal secretion, aching sinuses or reduced smell, all for at least 12 weeks during the last year**. Each individual symptom was a significant risk factor for multi-symptom asthma. *** P < 0.001.

**Figure 5:**
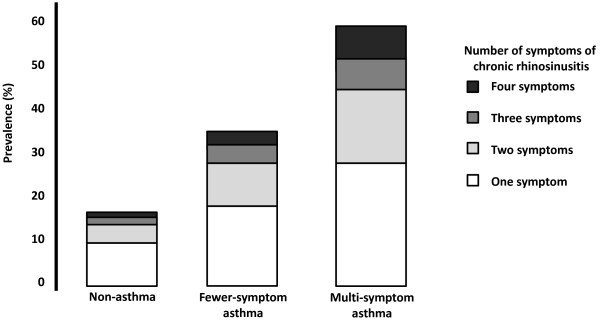
**Prevalence of the number of chronic rhinosinusitis symptoms in individuals with no asthma, fewer-symptom asthma, and multi-symptom asthma**. There was a higher frequency of multiple chronic rhinosinusitis symptoms in individuals with multi-symptom asthma.

Reports of nasal blockage, rhinorrhea, aching sinuses and/or reduced smell for at least 12 weeks during the last year, occurred with consistently higher frequencies in subjects with multi-symptom asthma compared with fewer-symptom asthma and non-asthma (Figure 4). The distribution of individuals with one or multiple symptoms of chronic rhinosinusitis is shown in Figure 5.

When applying a statistical model controlling for nasal blockage for at least 12 weeks over the last year slightly reduced the statistical effect of nasal blockage alone on the risk of having multi-symptom asthma (OR 2.05 versus 2.68).

## Discussion

Multi-symptom asthma is likely to describe a population with more severe disease, as night-time awakenings due to asthma were more common in this group. In addition, the importance of nasal symptoms as risk-factors for multi-symptom asthma is highlighted in this study. Nasal blockage and rhinorrhea, alone and together with allergic rhinitis, were more frequent in subjects with multi-symptom asthma, illustrating that the number of symptoms of rhinitis and severity of asthma are closely associated. Furthermore, symptoms of chronic rhinosinusitis, defined as nasal blockage, rhinorrhea, aching sinuses and/or reduced smell for at least 12 weeks during the last year were closely related to multi-symptom asthma.

When defining multi-symptom asthma, we included individuals reporting physician diagnosis of asthma, use of asthma medication, recurrent wheeze and attacks of shortness of breath, and one more asthma symptom, with the aim of identifying those with more intense disease activity. We suggest that a large component of subjects have a more severe degree of asthma, as they reported much higher frequency of night-time awakenings due to asthma compared with non-asthma and fewer symptom asthma groups. Furthermore, these subjects may represent a group that are "difficult to treat", as they reported several airway symptoms despite having access to asthma medication as required by the multi-symptom asthma definition. Defining severe asthma is not an easy task, as factors such as adherence to treatment, intensity, pathophysiological processes, and the presence of co-morbid conditions, which are clarified in an ATS/ERS statement [19] and the paper by Redel et al. [20], must be considered. In the present study, we have decided on using the term "multi-symptom asthma", as it is clearly definable from an epidemiological standpoint. Importantly, no previous attempt has been made to clearly define a group with more severe degree of asthma in previous large-scale population studies, which further illustrates the significance of the present approach. We suggest that our definition of multi-symptom asthma is an appropriate epidemiological tool to define this group of patients with substantially unmet needs [19].

The prevalence of rhinitis in the general population from the West Sweden Asthma Study, including reported allergic rhinitis, nasal blockage and rhinorrhea, was 37% [21]. However, in both the fewer- and multi-symptom asthma groups, the prevalence of allergic rhinitis increased to approximately 65%, which is in line with previous reports [22]. Thus, the presence of allergic rhinitis was not different between the two groups with different degree of asthma severity, whereas the presence of rhinitis is a clear risk factor for having asthma *per se*.

Importantly, the prevalence of nasal blockage and rhinorrhea was more than twice as high in the multi-symptom asthma population compared with fewer-symptom asthma, and approximately four times higher in the multi-symptom asthma population versus the non-asthma population. It is especially clear that the prevalence of several rhinitis symptoms was substantially higher in the multi-symptom asthma population, strongly arguing that number of nasal symptoms indeed is closely related to the severity of asthma, even though the prevalence of allergic rhinitis *per se *does not predict asthma severity. The two strongest risk factors for multi-symptom asthma versus fewer-symptom asthma identified in this study were nasal blockage and rhinorrhea. This is in agreement with clinically recruited cohorts [23], reporting that severe rhinitis is often associated with more severe asthma. Our study therefore strengthens these previous findings by confirming the close association between severity of rhinitis, and severity of asthma in general, in a random population, and, in addition, clarifying the true prevalence of these symptoms as well as the associations.

As nasal blockage is common in chronic rhinosinusitis, we determined the co-existence of symptoms of this disease with multi-symptom asthma. Indeed, any sign of chronic rhinosinusitis, defined as being present for more than 12 weeks a year, were more frequently reported in the population with multi-symptom asthma compared with both the non-asthma and fewer-symptom asthma groups. Interestingly, more than 60% of subjects with multi-symptom asthma had at least one sign of chronic rhinosinusitis, arguing that a close relationship exists between these conditions. Signs of chronic rhinosinusitis were also associated with multi-symptom asthma regardless of whether the individual reported allergic rhinitis or not, arguing that the allergic status of the individual may be unimportant for this interaction. However, clinical studies that investigate the sensitisation status in patients with signs of chronic rhinosinusitis and multi-symptom asthma are needed to confirm any such independence. An alternative hypothesis could be that infectious agents, including both viruses, bacteria and fungi, could interfere with both nasal symptoms and the severity of asthma [13].

In addition to the number of nasal symptoms, several other factors appear to distinguish the multi-symptom and fewer symptom asthma populations. A family history of allergy or both allergy and asthma increased the risk of having multi-symptom disease, although a family history of asthma did not clearly distinguish the two categories. In addition, old age, occupational exposure to gas, dust or fumes, and female gender are related to multi-symptom asthma, confirming the involvement of multiple factors for developing a more severe type of asthma. Previous risk-factor analyses of severe asthma have seldom been based on random samples, but rather on clinical cohorts, which lead to substantial selection bias in the analysis [15].

The strengths of the present study are that it has utilised well-validated epidemiological questionnaires, and it includes a very large random population, which contributes to high internal validity. The response rate was similar or higher than some other international studies of similar nature [24], albeit slightly lower than some other Swedish studies [25]. Importantly, a survey of those in the current study who did not respond to the questionnaire revealed no differences in prevalence of respiratory symptoms between responders and non-responders, and identical risk-factor profiles [25]. Nevertheless, a relative weakness of any study using postal questionnaires is that that all symptoms and diagnoses are self-reported, which introduces an uncertainty regarding the exact objective clinical diagnosis in each individual. However, the question "have you been told by a doctor that you have asthma" has proven to have very high specificity in Swedish samples [26]. Importantly, the questions used in this study about symptoms of chronic rhinosinusitis have recently been assessed, showing that answers were reasonably stable over time and between countries, were not influence by the presence of allergic rhinitis, and appeared suitable to determine prevalence of chronic rhinosinusitis in epidemiology (unpublished results, submitted for publication). Lastly, understanding and diagnosing chronic rhinosinusitis remains elusive, as epidemiological tools and clinical tools are poorly validated, and the pathophysiological processes are still poorly understood [27]. However, attempts to identify individuals with chronic rhinosinusitis in an epidemiological setting remains a high priority, and further phenotyping of these individuals will require detailed clinical investigations, which is beyond the scope of any large epidemiological approach to identify risk factors.

## Conclusions

This study describes the close association between the presence of several nasal symptoms and multi-symptom asthma, and underlines the difference in risk factor patterns for fewer- or multi symptom asthma. Unlike many previous studies that have evaluated the relationship between rhinitis and asthma severity, the present study is based on a very large, randomly-selected population, which substantially increases the validity of the results. Indeed, a large survey, such as the West Sweden Asthma Study, is required to achieve sufficient power to identify associations and risk factors in different disease sub-groups, such as the multi-symptom asthma group. The observed link between the extent of nasal symptoms and the presence of multi-symptom asthma, further emphasizes the importance that physicians consider the presence of asthma in patients who present with nasal symptoms, and vice versa.

## Abbreviations

OR: odds ratios, 95% CI: 95% confidence interval

## Competing interests

Jan Lötvall has received consultancy and speaker fees from AstraZeneca, GlaxoSmithKline, MSD/Merck, Novartis, and Schering-Plough.

## Authors' contributions

JL and BL conceived the work; LE performed the analyses. JL and LE wrote the core of the manuscript. All authors contributed to the discussion. All the authors read and approved the final manuscript.
